# Clinical Issues in the Prophylaxis, Diagnosis, and Treatment of Anthrax

**DOI:** 10.3201/eid0802.01-0521

**Published:** 2002-02

**Authors:** David M. Bell, Phyllis E. Kozarsky, David S. Stephens

**Affiliations:** Centers for Disease Control and Prevention, Atlanta, Georgia, USA

On November 18, 2001, a meeting was held at the Centers for Disease Control and Prevention (CDC), Atlanta, Georgia, to discuss the prophylaxis, diagnosis, and treatment of anthrax. Participants included clinicians and health department personnel from areas where anthrax cases were identified, infectious disease experts, representatives of professional societies, and experts from federal agencies. A patient recovering from inhalational anthrax also described her illness. The following is a summary of the presentations and discussion.

## Prophylaxis^1^

Ciprofloxacin, doxycycline, and penicillin G procaine have been approved by the Food and Drug Administration (FDA) for prophylaxis of inhalational *Bacillus anthracis* infection, on the basis of efficacy data in monkeys and pharmacokinetic, pharmacodynamic, and safety considerations (*1*–*3*). During the recent bioterrorist attacks, interim CDC recommendations for anthrax prophylaxis include ciprofloxacin or doxycycline; amoxicillin (in three daily doses) is an option for children and pregnant or lactating women exposed to strains susceptible to penicillin (*4*–*6*), to avoid potential toxicity of quinolones and tetracyclines. Amoxicillin is not widely recommended as a first-line prophylactic agent, however, because of lack of FDA approval, lack of data regarding efficacy, and uncertainty about the drug’s ability to achieve adequate therapeutic levels at standard doses.

The optimal duration of prophylaxis is uncertain; however, 60 days was recommended, primarily on the basis of animal studies of anthrax deaths and spore clearance after exposure. The possible need for longer prophylaxis and vaccine use was discussed. In monkeys after aerosol challenge, an estimated 0.5%-1% of spores remained at 75 days and traces were present at 100 days; delaying prophylaxis up to 20 days after exposure prolonged the incubation period without reducing disease risk (*7*). In one human case during the Sverdlovsk outbreak (former Soviet Union, 1979), anthrax developed 43 days after spores were released into the atmosphere (time of exposure unknown) (*2*,*8*). When prophylaxis is delayed or intermittent, several experts recommended a total of 60 days of therapy. (On December 18, the Department of Health and Human Services announced additional options for prophylaxis of inhalational anthrax for persons who wish to take extra precautions, especially those whose exposure may have been high. Three options are now offered: 1) 60 days of antibiotic prophylaxis; 2) 100 days of antibiotic prophylaxis, and 3) 100 days of antibiotic prophylaxis, plus anthrax vaccine as investigational postexposure treatment [3 doses over a 4-week period] [*9*].)

The need for prophylaxis is determined by public health officials on the basis of an epidemiologic investigation. Prophylaxis is indicated for persons exposed to an airspace contaminated with aerosolized *B. anthracis*. Prophylaxis is not indicated for health-care and mortuary workers who care for patients or attend to corpses using standard precautions, for persons who handle or open mail in the absence of a credible threat, or for prevention of cutaneous anthrax (*10*).

Successful implementation of mass prophylaxis requires clarity of public health intent and communication, as well as coordination and collaboration. A well-communicated policy on who receives prophylaxis and with which drugs is essential. Agency spokespersons, local health-care providers, employers, and employee organizations (e.g., labor unions) should be familiar with the policy. Local or regional task forces may be helpful in planning and communicating public health policy, and resolving jurisdictional issues. Prophylaxis teams should be predesignated to function around the clock. Team members should have contingency plans for personal needs (e.g., child care). Issues for the point of prophylaxis distribution include layout and managing of traffic flow; security; availability of medical and office supplies, antibiotic and disease fact sheets, multilingual staff, and mental health counselors; legal needs (e.g., for a physician to write orders); and plans for follow-up, including assessment of adherence, illness, and possible drug adverse effects. Collaboration among health departments, health-care delivery organizations, and clinicians is important. In the 2001 outbreak, some patients with possible drug side effects were refused appointments by their private physicians and were referred back to the health department.

Anthrax prophylaxis issues needing further consideration or research include efficacy of additional drugs, optimal duration of prophylaxis, usefulness of a loading dose, safety of prolonged drug use (especially in children and pregnant women), concomitant use of vaccine or antitoxin, level of infectious dose, and definition of high-risk exposure (e.g., according to particle size or degree of environmental contamination).

## Clinical Recognition and Diagnosis^2^

Twenty-two confirmed or suspected cases (11 confirmed inhalational; 7 confirmed and 4 suspected cutaneous) were identified in the 2001 outbreak of bioterrorism-related anthrax. Cases were reported from Florida, New York, New Jersey, the District of Columbia, and Connecticut.

### Inhalational Anthrax

Of the 11 patients with inhalational anthrax, 9 (and possibly all 11) are believed to have been exposed to mail containing or contaminated with *B. anthracis* spores. Median age was 56 years (range 43-94 years). Average incubation from known exposure to symptoms was 4 days (range 4-6 days). Fever, chills, drenching sweats, profound fatigue, minimally productive cough, nausea or vomiting, and chest discomfort were symptoms reported by most patients. Rhinorrhea and productive cough were uncommon. Chest X-ray at initial examination showed mediastinal widening, paratracheal fullness, hilar fullness, and pleural effusions or infiltrates or both, but in some patients these initial findings were subtle. Pleural effusions were a complication in all 11 patients; among all 8 patients who had not received antibiotics, *B. anthracis* grew in blood cultures drawn at initial examination. Six (55%) of 11 patients have survived with aggressive supportive care and multidrug antibiotic regimens including a fluoroquinolone (*11*).

The differential diagnosis of inhalational anthrax versus influenzalike illness is challenging. Respiratory viruses, including influenza, are common causes of influenzalike illness and tend to circulate in winter. These viruses are readily communicable, in contrast to anthrax, which is not spread from person to person. A history of influenza vaccination is not helpful in evaluating the likelihood of anthrax. Influenzalike illnesses have many causes besides influenza viruses, and influenza vaccine is not 100% effective. Unlike patients with inhalational anthrax, adults with influenza or other viral respiratory illnesses do not usually have shortness of breath and vomiting but often have sore throat or rhinorrhea. Rapid identification tests for influenza are available but vary widely in sensitivity.

In the current climate, emergency department and primary-care physicians should maintain a high index of suspicion for inhalational anthrax. Complicating diagnosis is the fact that patients initially may not appear very ill (*11*). A careful history with assessment of epidemiologic risk factors for anthrax (e.g., working for the postal service) should be obtained. Communication between clinicians and health authorities is critical for obtaining up-to-date assistance with diagnosis and management.

The classic chest X-ray findings—widened mediastinum or pleural effusions—may be subtle or absent on initial medical evaluation. In addition, these radiographic findings are not unique to anthrax: histoplasmosis, sarcoidosis, tuberculosis, and lymphoma, for example, are included in the differential diagnosis. A chest computed tomography scan is helpful in detecting hemorrhagic mediastinal lymph nodes and edema, peribronchial thickening, and pleural effusions, findings seen in patients with inhalational anthrax. Hyperdense mediastinal and hilar adenopathy plus mediastinal edema suggest anthrax. The hemorrhagic pleural effusions of inhalational anthrax typically increase during hospitalization.

Blood cultures and *B. anthracis*-specific polymerase chain reaction (PCR) of sterile fluids (e.g., blood and pleural fluid) are important in the diagnosis of inhalational anthrax. Serologic testing has also been valuable. An enzyme-linked immunosorbent assay (ELISA) to detect immunoglobulin (Ig) G response to *B. anthracis* protective antigen (PA) is highly sensitive (detects 98.6% of true positives) but is only approximately 80% specific. To improve specificity, a PA-competitive inhibition ELISA is used as a second, confirmatory step. Preliminary studies indicate that specific IgG anti-PA antibody can be detected as early as 10 days, but peak IgG may not be seen until 40 days after onset of symptoms.

Immunohistochemical examination of pleural fluid or transbronchial biopsy specimen, using antibodies to *B. anthracis* cell wall and capsule, also has an important role in the diagnosis of inhalational anthrax, especially in patients who have received prior antibiotics. Immunohistochemical examination can detect intact bacilli or *B. anthracis* antigens. PCR, serologic tests, and immunohistochemical tests are currently available at CDC or at certain laboratories in the Laboratory Response Network (LRN).

### Cutaneous Anthrax

Seven confirmed and four suspected cases of cutaneous anthrax were identified during the 2001 outbreak. Skin trauma was not associated with these cases of cutaneous anthrax. Exposure to contaminated mail was the apparent source of infection in all patients. The incubation period after exposure ranged from 1 to10 days. The initial symptom was often a pruritic papule resembling an insect bite. The papules vesiculated, with some becoming hemorrhagic. The vesicles ruptured to form depressed ulcers, often with local edema, ultimately forming dry eschars. These stages occur regardless of antibiotic therapy. The differential diagnosis of cutaneous anthrax includes brown recluse spider bite, ecthyma, ulceroglandular tularemia, accidental vaccinia, and necrotic herpes simplex. Cutaneous anthrax is painless, does not include rash, and results in a black eschar. Patients with cutaneous anthrax may have fever, extensive edema, and other systemic signs.

Gram stain and culture of the lesion are recommended; however, prior antibiotic treatment rapidly renders the infected site culture-negative for *B. anthracis*. Serologic testing and punch biopsy at the edge of the lesion, examined by silver staining and immunohistochemical testing, are useful in diagnosing cutaneous anthrax in patients who have received antibiotic therapy.

Clinical recognition and diagnosis issues needing further consideration and research include rapid, reliable, and readily available detection methods (e.g., PCR and antigen detection); education and ready access to information for clinicians regarding anthrax clinical features and risk stratification; recognition of anthrax in children; and the role of serologic testing in the diagnosis and management of both inhalational and cutaneous anthrax.

## Treatment^3^

Treatment recommendations for anthrax infections have been based on historical information and limited data from animals (nonhuman primates), as well as in vitro findings. Susceptibility testing of 65 historical isolates was performed at CDC. In the absence of published guidelines for testing for *B. anthracis*, the standard National Committee for Clinical Laboratory Standards broth microdilution method was used with staphylococcal breakpoints. These 65 isolates and all those associated with the 2001 outbreak were sensitive to the quinolones, rifampin, tetracycline, vancomycin, imipenem, meropenem, chloramphenicol, clindamycin, and the aminoglycosides. The isolates have intermediate-range susceptibility to the macrolides but are resistant to extended-spectrum cephalosporins, including third-generation agents (e.g., ceftriaxone), and to trimethoprim-sulfamethoxazole (*12*).

The decision regarding the use of penicillins, the drugs historically used for treatment and prophylaxis of anthrax, is complicated. An inhibition assay shows beta-lactamase activity at low levels in the isolates. Genomic sequence data show two beta-lactamases: a potential penicillinase (class A) and a cephalosporinase (class B), which is expressed. Concern about the use of penicillin arises because an inducible penicillinase could be activated in the face of treatment with beta-lactams, particularly if the number of organisms present is high, as appears typical with inhalational disease. Concerns have also been raised about the poor penetration of beta-lactams into macrophages, the site where *B. anthracis* spores germinate.

Ciprofloxacin has been recommended on the basis of in vivo (animal) findings; other quinolones have not been studied in the primate model. Doxycycline, another first-line agent, should not be used if meningitis is suspected because of its lack of adequate central nervous system penetration. Bacteremic patients are often initially treated with a multidrug regimen to which an offending organism is presumed sensitive; this treatment allows empiric coverage for other pathogens. Thus, the recommendation for initial treatment of inhalational anthrax is a multidrug regimen of either ciprofloxacin or doxycycline along with one or more agents to which the organism is typically sensitive. After susceptibility testing and clinical improvement, the regimen may be altered. The drugs of choice for treatment of cutaneous disease are also ciprofloxacin or doxycycline. A penicillin such as amoxicillin or amoxacillin/clavulanic acid may be used to complete the course if susceptibility testing is supportive.

On the basis of risk for the inhalational form of the disease, cases of both inhalational and cutaneous anthrax associated with the 2001 outbreak are being treated with 60 days of antibiotics. Although zoonotic cutaneous anthrax is treated with a 7- to 10-day regimen, inhaled spores can remain latent for extended periods.

Two months after the 2001 outbreak, 6 of 11 patients with inhalational anthrax had survived. Keys to successful management appear to be early institution of antibiotics and aggressive supportive care. Chest tube drainage of the recurrent pleural effusions, which are typically hemorrhagic, often leads to dramatic clinical improvement. Because these effusions tend to reaccumulate rapidly, insertion of a chest tube or tubes has been beneficial.

Anthrax treatment issues meriting further consideration relate to adjunctive therapies. Clindamycin has been suggested to have antitoxin properties (as in the treatment of toxic shock associated with group A streptococci, *Staphylococcus aureus,* and *Clostridium* infections). Steroids have been used to control the edema of cutaneous disease and have been suggested for the treatment of meningitis or substantial mediastinal edema (*13*). Other antitoxin agents investigated in vitro include angiotensin-converting enzyme inhibitors, calcium channel blockers, and tumor necrosis factor inhibitors. Specific anthrax IgG antisera, collected from military or other vaccinees, may be an adjunct, as well as administration of the vaccine itself.

## Footnotes

1 Presenters: David Ashford, David Bell, Susan Blank, Eddy Bresnitz, M. Dianne Murphy, Bradley Perkins, Larry Siegel, and Steven Wiersma.

2 Presenters: Sharon Balter, Carolyn Bridges, James Earls, John Jernigan, Michael Martin, Thom Mayer, Thomas McGovern, Carlos Omenaca, David Stephens, Martin Topiel, and Sherif Zaki.

3 Presenters: John Jernigan, Phyllis Kozarsky, Carlos Omenaca, David Stephens, and Fred Tenover.
